# Integrative multimodal treatment approach for patients suffering from Post-COVID syndrome: a study based on qualitative interviews with individuals participating in an 11-week day clinic program

**DOI:** 10.3389/fpubh.2025.1688592

**Published:** 2025-12-03

**Authors:** Christine Uecker, Christoph Schlee, Sandra Utz, Sarah Schmid, Jost Langhorst

**Affiliations:** 1 Department of Integrative Medicine, Medical Faculty, University of Duisburg-Essen, Essen, Germany; 2 Department of Internal and Integrative Medicine, Sozialstiftung Bamberg, Bamberg, Germany; 3 Institute for Sociology, University of Bamberg, Bamberg, Germany

**Keywords:** Post-COVID syndrome, fatigue, mind–body-medicine, integrative medicine, MICOM day clinic, stress reduction, qualitative study

## Abstract

**Background:**

Post-COVID syndrome is a complex condition affecting patients after SARS-CoV-2 infection. It is characterized by fatigue, pain, reduced resilience, and quality of life. Severe cases resemble myalgic encephalomyelitis/chronic fatigue syndrome (ME/CFS), associated with post-exertional malaise (PEM). Due to the unknown etiology and lack of curative options, multimodal therapy offers a particularly promising approach to symptom management.

**Methods:**

This qualitative interview study was part of a mixed-methods project conducted in 2022–2023, which investigated the effects of a multimodal integrative day clinic approach (MICOM day clinic) on the symptoms and general well-being of individuals with Post-COVID syndrome. The aim of the qualitative sub-study was to gain a deeper understanding of the individual perspectives of patients on their experiences of the illness and the perceived changes reported by 20 intervention group participants after completing the 11-week (66-h) multimodal program. Interview data had been analyzed based on reflexive thematic analysis using MAXQDA software.

**Results:**

Participants reported significant impairments in daily life and work capacity, accompanied by high psychological burden. The multimodal program led to improvements in physical and mental symptoms, overall well-being, and self-efficacy. Patients also reported long-term lifestyle changes in areas such as nutrition, stress regulation, and performance-oriented physical activity.

**Conclusion:**

Post-COVID syndrome poses ongoing challenges for individuals and healthcare systems. Multimodal integrative treatment can effectively reduce symptoms, enhance coping strategies, and promote sustainable self-care.

**Clinical trial registration:**

Clinicaltrials.gov, identifier NCT05630378.

## 1 Introduction

At the end of 2019, the first cases of a novel coronavirus infection appeared in China and in January 2020, the first official case of infection with the SARS-CoV-2 virus was documented in Germany (1). As of July 2025, around 778.5 million cases worldwide have been documented and nearly 7.1 million people have died from or with the infection (no clear other cause of death) (2, 3). The international health emergency was officially lifted by the WHO in May 2023 and the pandemic phase was declared over. Nevertheless, many people are suffering from sequelae of the infection today. If symptoms persist for more than four weeks and up to three months after the acute infection, this is referred to as Long-COVID syndrome (4, 5). Post-COVID syndrome (PCS) or Post-COVID condition (PCC) is according to WHO defined as presence of symptoms for more than 12 weeks after the initial onset of infection, last at least two months, with no other explanation for them. The course of the condition may be persistent, recurrent or fluctuating (6). The prevalence of PCS is reported differently (depending on the way of assessment), it is estimated that at least 10% of those infected experience ongoing symptoms (7, 8). A systematic review and meta-analysis including 48 studies worldwide found a pooled prevalence of 41.79% overall and 46.28% in Europe for Post-COVID syndrome, whereby the prevalence for PCS in females was higher than in males (9). The German medical guideline assumes at least 3–6% of infected persons suffering from persistent symptoms after 3 months (4).

PCS is characterized by a variety of non-specific symptoms affecting various organ systems. The majority of patients experience fatigue and impaired performance, as well as muscle/joint pain, headaches, impaired concentration and word-finding difficulties (also called “brain fog”), palpitations, sleep disturbances, dizziness, hypersensitivity and other symptoms (4, 10, 11). The presence of post-exertional malaise (PEM) indicates Myalgic Encephalomyelitis (ME)/Chronic Fatigue Syndrome (CFS), which can also occur after other viral infections such as the Epstein–Barr virus, influenza or herpes virus. A proportion of PCS patients also develop ME/CFS (12). Overall, PCS leads to a significant impairment in patients’ everyday lives and a reduction in their health-related quality of life (13–17). PCS patients often experience long periods of sick leave and limited ability to work (18, 19).

Like in ME/CFS the pathogenesis of PCS is still not fully understood and causal (drug or non-drug) therapies based on the results of randomized controlled trials are scarcely available. The updated medical guideline on Post-COVID syndrome recommends multimodal and interdisciplinary treatment (4). In addition to the symptomatic therapy of accompanying symptoms, interventions for energy and stress management as well as psychosocial support are generally recommended (20). With regard to pain, dependent on the type of pain, multimodal treatment strategies including non-pharmacological therapies (e.g., the use of coping strategies like relaxation techniques, exercise programs and lifestyle interventions) should complement pharmacological pain medication (4, 20, 21).

These therapy recommendations align with the EUROMENE guideline for ME/CFS treatment, which also emphasizes the importance of non-pharmacological therapies, such as relaxation, meditation/mindfulness, and manual therapies. Physiotherapy and acupuncture can reduce pain, while relaxation techniques can positively impact sleep disorders and stress or anxiety (22, 23). An anti-inflammatory diet within a multimodal approach is also considered potentially beneficial in improving fatigue symptoms (22, 24). While also exercise therapy can alleviate fatigue (25), caution is advised for patients with post-exertional malaise (PEM): they require individually adapted exercise programs to avoid deterioration. This can be prevented through well-dosed, personalized physical activity, i.e., activity management (“pacing”) (4, 26). According to a systematic review, mind–body interventions appear to have positive effects on fatigue, anxiety/depression, and mental functioning in ME/CFS patients (27).

With regard to pharmacological therapies, there are some off-label therapy approaches (e.g., anti-inflammatory, antiviral, immunomodulatory drugs), which might be effective, but need to be approved by randomized controlled trials to ensure their safety and efficacy (28, 29). Furthermore, a range of additional supportive approaches may be employed to exert a favorable influence on the progression of the disease and to ameliorate symptoms. For instance, the administration of vitamins to enhance immune system function could be advantageous. Vitamin C has been demonstrated to function as an antioxidant, to manifest anti-inflammatory and immunomodulating properties, and to enhance endothelial function (30). The role of an adequate supply of vitamin D has also been discussed. This could at least have a preventative effect of late effects especially in case of a vitamin D deficiency but needs to be tested further (31, 32). Phytotherapeutics such as *Rhodiola rosea*, which is used as an adaptogen, can alleviate stress-induced fatigue (33, 34).

The role of the intestinal microbiome in the development and progression of PCS is also a topic of discussion: an appropriate diet (conducive to the gut microbiome), is believed to have a beneficial effect on the course of the disease, given the established link between the gut microbiome and the immune system (35, 36).

The use of mild water-filtered infrared-A whole-body hyperthermia (WBH) from the field of hydro- and thermotherapy led to a reduction of pain in fibromyalgia patients (37, 38). This treatment modality has the capacity to stimulate and activate the immune system, thereby augmenting blood circulation and tissue perfusion (39–41), which is particularly interesting since a reduced/restricted microcirculation in the tissue is discussed as one possible cause of Post-COVID symptoms (4). To date, the number of studies on the use of WBH in patients with Post-COVID syndrome is limited. A study conducted by Vagedes et al. (42) reported the observation of positive effects on fatigue in patients diagnosed with PCS when WBH was integrated within a multimodal framework encompassing integrative therapy.

The treatment approaches presented above are predominantly used in Traditional, Complementary and Integrative Medicine (TCIM) and are also part of the comprehensive, integrative therapy concept on which this study is based. The Department for Internal and Integrative Medicine in Bamberg treats chronically ill patients with a multimodal concept based on mind–body medicine and elements of traditional European medicine following Sebastian Kneipp (1821–1897) (43). The underlying integrative concept was developed in Essen (MICOM - mind–body medicine in integrative and complementary medicine) (44). In Bamberg it is applied in an inpatient setting and a day clinic setting and includes various therapy options and techniques: mind–body medicine, body awareness, mindfulness-based stress management and relaxation techniques, healthy nutrition, exercise therapy (e.g., endurance and strength), phytotherapy, hydro- and thermotherapy, and naturopathic self-help strategies. Patients with PCS who are suffering from pronounced fatigue with or without muscle and joint pain and other symptoms for more than three months after infection are treated as inpatients, day clinic patients and outpatients in Bamberg. In contrast to more individually tailored medical treatment during inpatient care, the focus of the day clinic is on teaching self-help strategies and guidance for lifestyle modifications in a group setting. A healthcare research study on the inpatient treatment of PCS in Bamberg showed that a multimodal 10- to 14-day inpatient treatment led to a significant reduction in fatigue and an increase in the subjectively perceived ability to work at the time of discharge (45).

The positive effect of the integrative multimodal concept in form of a day clinic was evaluated before with regard to other chronically ill patient groups, where it is also assumed that the reduction of stress in combination with a healthy, mindfulness-based lifestyle has positive effects on the course of the disease. Patients suffering from ulcerative colitis or Crohn’s disease - both are chronic inflammatory bowel diseases in which, among other factors, also immunological processes play a role - showed positive effects on symptoms, self-efficacy and quality of life after participation in an integrative day clinic program (46–49).

The WBH was evaluated in a randomized controlled trial in patients with fibromyalgia syndrome – a disease which is characterized by the main symptoms of pain and fatigue. Patients experienced a significant reduction of pain symptoms after the application of up to 6 WBH (37).

Based on the current state of research, this qualitative study aims to improve the understanding of Post-COVID syndrome and how to deal with it. The study examines a multimodal approach which combines various therapeutic elements as a potential treatment option for the large group of PCS patients. Given the prevailing lack of understanding and the individuality and complexity of the Post-COVID condition, investigating the perspectives and experiences of PCS patients in the context of this approach can contribute to a greater understanding of PCS and the field. Therefore, the following research questions aimed to be answered: How do participants experience Post-COVID syndrome in everyday life? What measures were taken before participating in the day clinic, and what support did they receive from medical professionals? What changes are reported as a result of participation in the day clinic program?

## 2 Materials and methods

### 2.1 Study design and intervention

**Figure 1:**
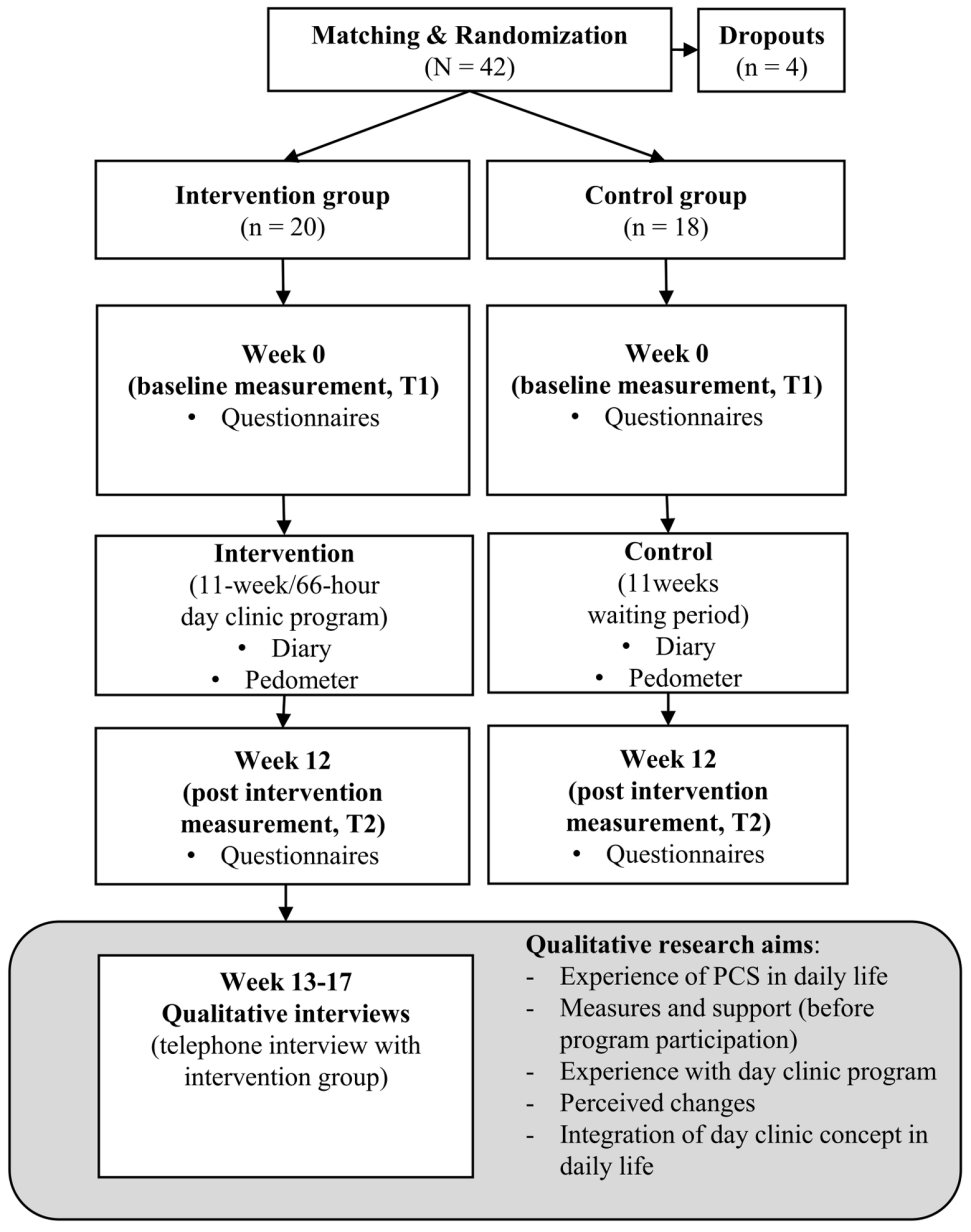
Flow-chart: mixed methods study design.

This paper presents the results of a qualitative study which was designed as part of a mixed-methods research project based on a randomized controlled trial on a multimodal integrative approach for Post-COVID syndrome (Figure 1). The presented study can be described as a qualitative study that is embedded or nested within an RCT approach (50). The design and methodological approach of the study was based on two qualitative studies already conducted in our research group as part of mixed-methods projects (46–49). The overall study was conducted at the Department for Internal and Integrative Medicine at Bamberg hospital in 2022/2023 and aimed to evaluate the effects of an 11-week (66-h) multimodal integrative day clinic, treatment program on the symptoms (particularly fatigue and pain) and quality of life of patients with Post-COVID syndrome.

In the underlying randomized controlled study the following in- and exclusion criteria for participants were applied to the overall study: Patients aged 18–75 years with a confirmed diagnosis of Post-COVID syndrome, i.e., persisting symptoms for more than three months after initial infection with SARS-CoV2-virus, presence of fatigue with or without myalgia, and who signed an informed consent form were eligible for participation. Main exclusion criteria were critical illness and intensive care following acute SARS-CoV-2 infection, since the day clinic concept is not suitable for severely affected patients who are probably unable to attend the weekly six-hour sessions over a period of 11 weeks. Also, patients with severe somatic, cardiovascular, pneumological, rheumatic, endocrine or neurological concomitant diseases (accompanied by cognitive disorders), severe liver or kidney diseases or severe psychiatric illness were excluded from the study. In addition, patients with contraindications for WBH treatment were excluded from participation (please see Suppl. 1 for detailed information on in−/exclusion criteria).

A variety of strategies were employed to recruit the study participants. In addition to the information provided on our clinic’s website, flyers were disseminated in outpatient medical practices. Two brief reports concerning the study were published in the local daily press. Additionally, we reached out to self-help groups for further insights.

**Figure 2:**
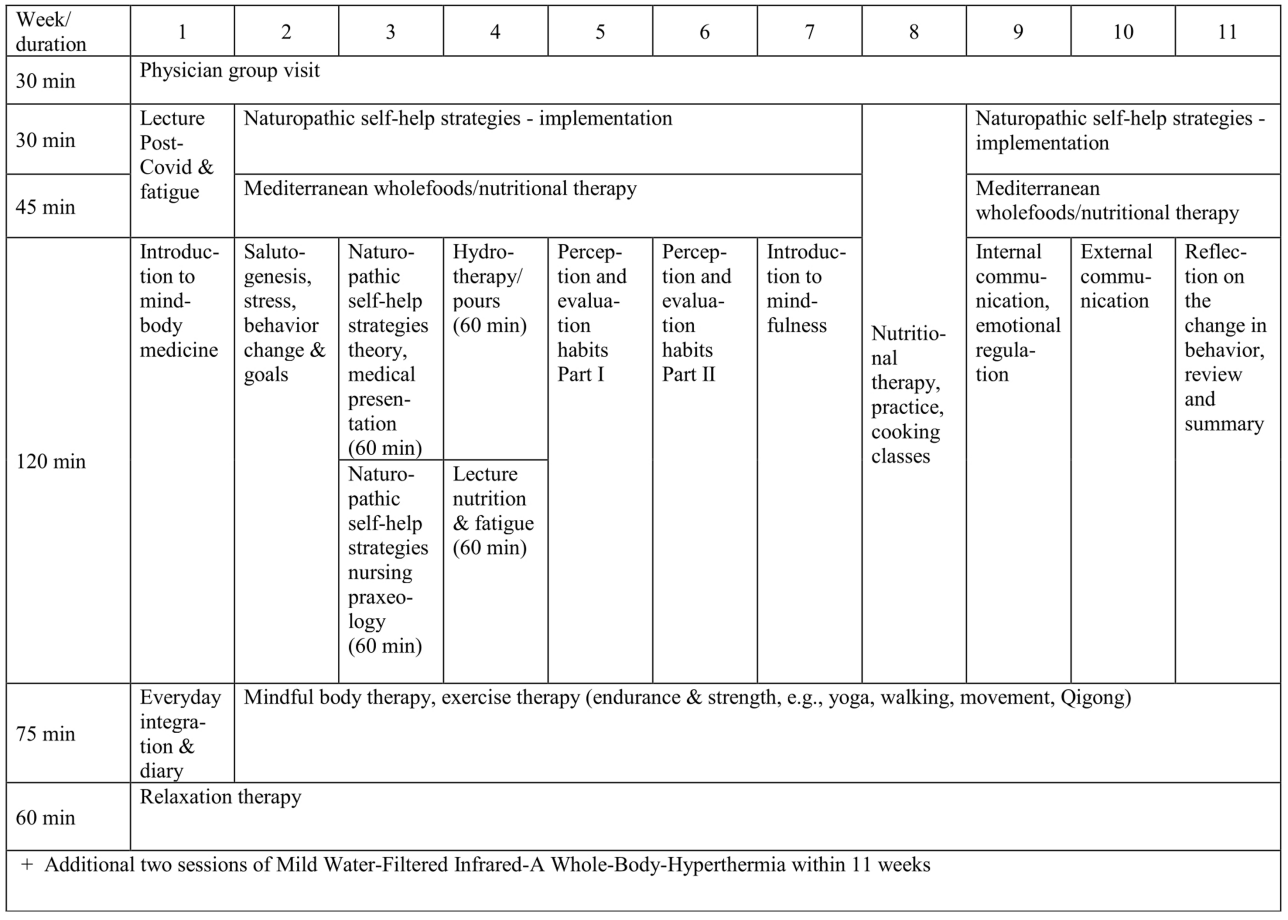
Exemplary schedule and content of the day clinic program [adapted from Paul et al. (52)].

The RCT consisted of an intervention group (*n* = 20) and a waiting control group (*n* = 18). Patients were randomized at a ratio of 1:1 into one of the groups, with stratification by sex, age, and general fatigue (measured by MFI-20 at baselineT1) (51). The treatment group received an 11-week day clinic (one day per week, 6 h per day, 66 h in total) and, if suitable, treatment with WBH twice during this period (Figure 2). The waiting control group had the opportunity to receive the intervention after their waiting period had ended and they had completed their participation in the study. However, they received no specified treatment during the intervention group’s treatment period. Both groups kept a diary documenting energy level, pain, quality of sleep, medication and concomitant therapies, and wore a pedometer to record their physical activity. For the quantitative research aim (RCT), which is not addressed in this paper, patients in both groups were given the same set of questionnaires at weeks 0 (T1) and 12 (T2) to measure, among others, fatigue of the patients (MFI). The results of the quantitative data assessed by several questionnaires will be published separately. The qualitative sub-study (only intervention group) embedded in this RCT, which constitutes the study described in this article, aims to understand patients’ subjective experiences with PCS in everyday life and how they cope with it. The focus is on the perceived changes resulting from the intervention. Therefore, only the intervention group was interviewed.

The participants of the intervention group were provided with a combination of theoretical information and practical training (52). The following therapeutic modalities were addressed in the day clinic: mindfulness-based stress reduction (e.g., relaxation and body scan), naturopathic self-help strategies (e.g., wraps and pads, Kneipp hydrotherapy), exercise therapy (e.g., endurance and strength), Yoga, Qigong and walking. Furthermore, the program included nutritional therapy and mild water-filtered infrared-A cooking classes based on a plant-based Mediterranean whole foods diet. In addition, patients received a maximum of two sessions of WBH over the course of 11 weeks, with the aim of reaching a core body temperature plateau of 38.5 °C for a minimum of 15 min. Moreover, the participants were given audio-guided relaxation and mindfulness exercises, as well as print materials that they could use at home to support the implementation of program elements in their daily lives. (52).

All patients in the intervention group (*n* = 20) were interviewed at weeks 13–17, in the majority of cases, approximately two weeks after the day clinic intervention had ended. The interview period (data collection period) occurred from August 2022 to March 2023. The aim was to get a deeper understanding of patients’ experiences of their illness and the impact of attending the day clinic on their illness and daily life over the past few months.

### 2.2 Semi-structured interviews

The objective of the present study was to collect a variety of perceptions and experiences and to analyze them for relevant patterns (53, 54). Therefore, a semi-structured interview approach with an interview guideline was employed to ensure specific topics were addressed and to consider prior knowledge and theoretical considerations (deductive elements). The structure of the guideline with its topics provided a basis for comparison for the interviews regarding the relevant research topics (55). However, with a general openness and flexibility in the approach to conducting the interviews, new insights as well as individual topics relevant to the participants can be considered (inductive elements) (56).

The interview guideline was based on previous studies conducted by our research team on participants’ experiences with a comparable day clinic concept (46, 47) and covered the following main topics: (1) experiences with the disease (Post-COVID syndrome) in everyday life (incl. Impact, duration, change over time); (2) medical care, previous measures against the disease and motives and expectations regarding participation in the day clinic program; (3) effort of participation (incl. Compatibility with employment/everyday tasks); (4) satisfaction with the program; (5) transfer of program content into everyday life (incl. Changes compared to before participation, integration of program content, hurdles to integration); (6) perception of effectiveness (incl. Health changes compared to before the program, future prospects).

The interviews were conducted by telephone. With patient informed consent, the interviews were recorded (audio file only), transcribed, and then anonymized. The interviewers were two sociologists who were experts with many years of experience conducting and analyzing qualitative interviews. The research process is characterized by a high degree of openness, and there was no prior contact with the interviewees. The researchers pursued an open data collection approach with open-ended questions, which gave the participants the opportunity to introduce new topics and assign their own meanings to the subject from their perspective. No theoretical assumptions were made in advance; the focus was on the participants’ perspectives instead. After the interviews, the interviewers wrote field notes to record the situation and any special occurrences or problems throughout the interview. No problems arose in this regard that affected the quality of the interviews.

### 2.3 Data analysis

To examine the relevant themes and patterns in the subjective meanings of the patients, the analysis was conducted using a reflexive thematic analysis approach (54, 57). The analysis was carried out with the support of MAXQDA software (58).

In the analysis both data-driven (inductive) and theory-driven (deductive) coding approaches were employed. This approach allowed for the appropriate consideration of both the empirical data and the underlying research questions and theoretical considerations. Since many aspects of Post-COVID syndrome remain unclear, a mainly inductive approach was chosen. The analysis was based on open coding. No code scheme was developed in advance. Instead, it was developed based on the data in the first step. Open coding was the basis for the analysis. In addition, categories were developed deductively, which were largely determined, guided, and structured by the research questions and the research objective (Table 1). Moreover, previous studies of the day clinic intervention by our research group were considered in this context, which served as a framework (46, 47). To ensure intersubjectivity and validity the results were discussed within the research team during the analysis (59, 60).

**Table 1:** Excerpt from coding scheme—main categories.

Main codes	Sub codes
Acute COVID 19 -infection	
	History of infection(s)
	Recovery/further course
Impact of PCS on everyday life	
	Physical level (symptoms)
	Psychological level
	Social level (contacts and activities)
	Ability to work (professional performance)
Medical care	
	Care provided by general practitioners
	Previous therapeutic measures
Experience with day clinic program	
	Overall concept
	Therapy elements
	Organizational aspects
Changes in daily life after participation	
	Physical level (symptoms)
	Behavioral level (lifestyle modifications, implementation of concept elements in everyday life)
	Social level (participation and activities)
	Psychological level (emotions, changes in attitudes, stress management and self-efficacy)

## 3 Results

### 3.1 Interview sample

The interviews with all 20 participants of the intervention group - all of them completed the intervention - were conducted after the end of the day clinic program via telephone. The average interview length was approximately 43 min, with a range of 28 to 70 min. The Sample comprised 17 women and three men (85% female, 15% male) with an age range of 23 to 66 years (M = 48.5 years; SD = 11.26). Regarding their educational level, eleven participants achieved lower secondary education and nine participants achieved higher secondary education. The participants also differed in terms of time since COVID-19 infection (range of 2 years, 4 months), and current employment status (Table 2). With regard to pre-existing conditions, some patients reported chronic pain diseases (e.g., arthrosis or other musculoskeletal pain, or migraine) and three patients each suffer from thyroid disease (e.g., Hashimoto), hypertension, allergies, or asthma. Almost all patients reported feeling worse after physical or mental exertion; post-exertional malaise is very pronounced in three patients. One patient was hospitalized during acute COVID-infection, two participants spent several weeks in a rehabilitation clinic due to their Post-COVID symptoms.

**Table 2:** Overview sample characteristics.

Patient	Sex	Age (at the time of interview)	Educational level	Duration PCS (approx. time since initial infection)	Current employment status/employability (at the time of the interview)
01	f	37	2	9 months	Reintegration in process, working 2 h/day
02	m	50	1	7 months	Start of reintegration process 6 weeks ago, currently working 30 h/week
03	f	45	2	10 months	On sick leave for 9 months, now working 4 h/week
04	f	57	2	9 months	Unemployed
05	f	51	2	1 year, 10 months	Employed, but reduction in working hours from 7 to 6 h/day (reduction from 35 to 30 h/week)
06	f	66	1	1 year, 9 months	Retired
07	m	57	1	1 year, 9 months	On sick leave for 9 months
08	f	39	2	1 year, 5 months	Currently unable to work, recurring sick leave periods since infection
09	f	30	2	9 months	Full-time work (remote)
10	f	54	2	1 year, 9 months	Full-time work
11	f	57	1	2 years, 4 months	Unemployed, recently resigned due to PCS
12	f	53	1	9 months	Sick leave for 9 months
13	f	54	1	1 year, 7 months	Sick leave for approx. 5 months (several former periods on sick leave and failed attempts at reintegration)
14	f	53	1	2 years	Employed and self-employed (40–45 h/week in total)
15	f	52	1	2 years	Currently in the process of reintegration, (several periods on sick leave)
16	f	35	1	11 months	On sick leave for 11 months
17	f	65	2	1 year, 2 months	Retired (for 4 months)
18	m	40	1	10 months	On sick leave for 10 months
19	f	52	1	1 year, 11 months	Working 4–5 h/week
20	f	23	2	10 months	Student

1 = lower secondary education, 2 = upper secondary education.

### 3.2 Symptoms and impact on everyday life before intervention

All participants in the day clinic program reported significant constraints due to the illness in everyday life. Their overall health condition was severely impaired by various symptoms, in particular by *fatigue*, and only a few participants were able to reasonably meet their professional and family obligations at the time of the day clinic start. In some cases, Post-COVID symptoms did not begin immediately after the acute infection; rather the condition worsened again after an initial improvement. Overall, the symptom picture in PCS was complex and heterogeneous. The participants reported severe physical and mental exhaustion and reduced resilience. They had to take frequent breaks during the day, even after minor exertion, and most participants were not able to work to the same extent than before infection.

*“After that time [i.e. acute infection], I was still very exhausted, I couldn't walk up the stairs, one floor at a time, I couldn't walk at all. And it just didn't get any better for a very, very long time.” (P 3, female, 45 years)*

*“And for me, the worst thing was the exhaustion, because out of seven days a week, I had maybe five or six days at the beginning when I couldn't do anything. Towards the end, there were three or four days where I was really totally exhausted and yes, I had to fight my way out of that.” (P 19, female, 52 years)*

Patients reported an increased need for sleep and a lack of energy upon waking. Sleep often did not lead to sufficient recovery. Some patients explicitly reported experiencing *post-exertional malaise (PEM).*

*“Once I've finished, I'll do the few things I have to do, and then I'll be completely exhausted again. I might go out in the city for a little while, but then I have to lie down for three hours before I can carry on.” (P 20, female, 23 years)*

Around half of the patients also reported *cognitive impairments* such as concentration problems, memory disorders or word-finding problems, which made it extremely difficult for them to carry out everyday tasks or professional tasks that they had previously performed easily and routinely. This leads to uncertainty and worry among those affected.

*“And then, these mental lapses […] I used to have all the zip codes in the area in my head. Then I couldn't remember our own. I really had to look them up on the internet. I couldn't get it right any more. I would sometimes sit at my desk, I'd been doing that job for 15 years. I no longer knew what I had to do. I had no idea what I had to do, it was - and it wasn't very strenuous work […]” (P 17, female, 65 years)*

In addition, five participants complained of persistent muscle and/or joint pain or headache, and five patients experienced shortness of breath or a feeling of pressure or tightness in the chest. Circulatory problems, heart stumbling and palpitations, dizziness, sleep disorders and anxiety were also mentioned as burdensome by some participants.

The symptoms mentioned were perceived as a significant reduction in quality of life by the patients, which in turn led to psychological complaints as well. Furthermore, the unpredictable progression of the illness partly resulted in feelings of dejection and a sad mood.

*“I notice it clearly. I notice it physically and I notice it mentally. I noticed it now, when I was incapacitated for two days, what a mental burden it is, because everyone says, yes, then chill out, rest, enjoy the fact that you can sleep. It's not enjoyable, you're just completely exhausted and can't do that/ So it's not relaxing either, even if you sleep there. It's not relaxing. And what it does to you psychologically when you've had it for weeks and months […]” (P 19, female, 52 years)*

At the time of the interview, only three participants were able to work to the same extent as before the infection. Several patients were in the reintegration phase, which means that they were in the process of gradually extending their daily working hours over a period of weeks. Some had permanently reduced working hours, had resigned voluntarily from their job as a result of their health issues or retired. Six participants were still on sick leave (see Table 2). Participants who maintained full-time employment further remarked that this was only made possible through their capacity to adapt their working conditions, e.g., working from home. The reduced *ability to work* is perceived as a significant burden by patients. Participants expressed feelings of empathy toward their colleagues, who were required to-compensate for their reduced performance. Also, financial imperatives the prolonged absence from work due to sick-leave is commonly associated with financial losses - can result in an augmented burden and increased stress.

*“Exactly, when I started, I think my reintegration [back to work] was just about to end, although I have to say that I actually ended it too early. But that was also for financial reasons. At some point I didn't want to feel the pressure of earning less money all the time and then I went through with it.” (P 19, female, 52 years)*

Patients reported that they also experience major restrictions in their family and social life due to their greatly reduced resilience. They were unable to enjoy leisure activities or care for their families as they did before the illness.

*“And that also really means no more cinema, no more theater, no more readings, even meeting friends somehow restricted, visits, walks, because I just didn't have enough strength anymore.” (P 11, female, 57 years)*

Some reported that they are sometimes confronted with little understanding by family, friends, and colleagues because others cannot empathize with their exhaustion after minor exertion. Patients themselves often find this difficult to accept, so they can understand that it is even more challenging for others to grasp.

*“Friends, family, yes, it's still difficult because they just can't understand it, how can you go from sleeping for eight hours, getting up and being tired, to put it bluntly. Or why do you now have breathing problems when you're sitting or standing, even though you haven't done anything.” (P 16, female, 35 years)*

Experiencing limitations and, above all, uncertainty about the further progression of the disease and recovery prospects can also lead to *psychological problems* for those affected. They partly feel listless, depressed, and anxious. Patients also suffer from being unable to fulfill their duties at home and work as they used to and they feel like a burden to others (family members, friends, and work colleagues).

*“For example, three weeks ago I had a colleague at work, because I only work four hours, and he had to finish my work. But he doesn't talk to me anymore, because in his eyes I'm a malingerer and I'm just faking it and so on.” (P 2, male, 51 years)*

### 3.3 Experience with healthcare system: primary care and therapies

The majority of patients felt that their general practitioner (GP) who was usually the first point of contact did not provide sufficient care for their illness. Although most GPs were understanding and tried to help patients by referring them to specialists (e.g., cardiologists, neurologists) to rule out organic causes, hardly any treatment options were offered apart from symptom-oriented treatments (e.g., pain medication, inhalation) and general measures such as taking vitamins, drinking lots of tea and resting. The repeated issuing of sick notes for limited periods of time was interpreted as a wait-and-see policy by the patients.

*“[…] because my family doctor, unfortunately, didn't know what to do. He put me on sick leave for a day or two, or sometimes a week, if I felt unwell. But he always shrugged his shoulders. He didn't really know.” (P 5, female, 51 years)*

Some patients interviewed felt that their GP was not taking them seriously or even had the impression that their GP suspected a psychosomatic condition or a mental illness.

*“My family doctor, who I otherwise hold in very, very high regard, didn't believe me. She didn't believe me, at least that's how I felt. […] And she saw the whole thing as psychosomatic. And she wanted me to consult a psychologist and thought that would solve my problems.” (P 8, female, 39 years)*

*“The fact that you're not taken seriously by some doctors or by your environment, by your employer, somehow you get into a deep phase or a deep hole and you think to yourself, yes, is it really true, am I perhaps just making it up, am I imagining it, what is it actually like?” (P 16, female, 35 years)*

Due to their pronounced symptoms of exhaustion, it was at the same time particularly stressful for patients to look for treatment options themselves, apply for an inpatient rehabilitation program, travel to more distant specialized PCS centers, etc. Therefore, patients very much welcomed the opportunity to take part in the multimodal program offered in this study.

### 3.4 Changes due to participation in the day clinic program

At the end of the day clinic, all participants reported various predominantly positive changes they had noticed as a result of participating. These changes were related to their symptoms, their attitudes toward health and well-being, and their behavior, i.e., modifications in lifestyle.

As described above, the participants in the study showed a variety of symptoms. All patients suffered from pronounced fatigue (inclusion criterion), and many patients additionally reported pain (muscle, joint and limb pain; headaches), concentration difficulties, circulatory problems and dizziness, breathing difficulties, sleep disturbances, and other symptoms. Eleven participants narrated improvements in symptoms as a result of attending the day clinic program: they reported reduced fatigue and pain and increased energy levels. Positive effects were also mentioned with regard to sleep disorders, anxiety, and shortness of breath. The improvement in symptoms directly impacted the ability to participate in social life again.

*“Mainly the headaches and the pain in my limbs and muscles [have improved]. So, the pain has become much less. They haven't gone, but they've become a lot less. […] It's improved, so I'm already less exhausted, but I'm still a long way from being fit or from where I was before.” (P 3, female, 45 years)*

In addition to improvement in symptoms, the participants mainly attached high importance to the changes at the *attitude* level toward illness and their overall well-being.

*“A lot has changed, the biggest thing that has changed is simply my mental attitude, I can say a thousand percent that if I hadn't done the study, I wouldn't be feeling so well in terms of my head and my own well-being. Maybe I would already be in a depression, I have to be honest.” (P 16, female, 35 years)*

They particularly reported that participating in the day clinic had broadened their perspective, making them aware of important correlations such as the link between stress and physical symptoms. Some only recognized this connection through the day clinic.

*“Of course, the whole thing has a lot to do with stress and with what perhaps happened before the illness, which I also became aware of through the day clinic. And simply dealing with stressful situations has also become much better as a result and I think it also has a lot to do with the fact that the symptoms have also improved.” (P 9, female, 30 years)*

One third of patients explicitly mentioned increased *self-efficacy* and the feeling that they could influence the course of their illness. The day clinic gave patients more confidence in their own strengths and enabled them to take better care of themselves. They no longer felt at the mercy of the disease, instead, they felt they could do something about it themselves, which supported the therapeutic success and improved their well-being.

*“I no longer experience the whole thing in such a way that I am no longer at the mercy of it. I know about the symptoms. I know that I have to take a break much earlier, take care of myself, much more than I did before.” (P 17, female, 65 years)*

The majority, 14 patients, also reported an enhanced awareness of their mind and body condition indicating an improved ability to recognize when their body or mind requires rest. So, they can more effectively assess their own resilience. In the event of experiencing the initial symptoms of stress, it is possible for individuals to utilize appropriate stress-relieving strategies. Such strategies, which are used by all participants after the day clinic, may include the following: leaving the situation, going outside, and performing breathing exercises. It is evident that there has been a marked improvement in their level of attentiveness and care toward themselves. Furthermore, there is a notable increase in their sense of calmness and relaxation.

*“To think about natural things again. Paying attention to my body. Paying attention to my breathing. Giving the body breaks when it really needs them instead of ignoring them.” (P 18, male, 40)*

This form of mindfulness is particularly useful for implementing *pacing* strategies. It enables patients to adapt their activity level to their individual capacity more effectively and take breaks as needed to avoid physical or mental overexertion. In this context, some participants found the pedometer a helpful tool for implementing the pacing strategy. Using this, the patients were able to maintain a more accurate perspective of their ambulatory activity and could take restorative breaks if necessary to avoid overexertion. This approach facilitated the establishment of a more precise understanding of their personal limits, while also enabling the observation of changes in their movement profile over time

*“I have a lot more confidence in my own strength again, so to speak. I've also got to know my body a bit better now thanks to the pedometer and perhaps I know how to assess my limits better.” (P 8, female, 39 years)*

Overall, the perceived improvements in symptoms and overall well-being led to patients feeling happier, enjoying life more and being more optimistic about the future and having more confidence in their own abilities and strengths.

*“And I simply have the hope and confidence that things will get better. And I think that/ the day clinic made a significant contribution to developing confidence, acceptance and compassion for myself.” (P 1, female, 37 years)*

*“And to learn not to give up and then perhaps fall into a mental hole, but, as I've just said, to try to accept it and then make the best of it in order to perhaps gradually improve your performance again.” (P 7, male 57 years)*

### 3.5 Behavioral level/integration of program elements into everyday life

The day clinic concept in which patients were encouraged to make active changes themselves rather than being treated as a primary care option, has been well received. All participants reported that they had integrated some of the techniques they had learned during the program into their everyday lives.

*“It was clear to see that the aim of the day clinic is not to treat you with these applications, but simply to show you how to help yourself and apply them at home. But it's really good when someone shows you something like this once a week and you can really experience the application.” (P 3, female, 45 years)*

They made specific lifestyle changes with regard to relaxation, stress reduction, exercise, and nutrition. At the behavioral level, the participants reported primarily a change in the way they deal with *stress*. By attending the day clinic, patients learnt and practized various strategies for alleviating stress and relaxation in everyday life. So, in particular, breathing exercises, yoga, Qigong, progressive muscle relaxation (PMR) and meditation are used in everyday life.

*“Because then, for example, I simply pay attention to my breathing for a short, very short time. Maybe just two or three minutes of a short kind of meditation. And then I notice how I can be more relaxed again.” (P 8, female, 39 years)*

Patients have also integrated exercise units, such as morning gymnastics and walking, into their daily routine. They reported these morning exercises and walks in nature to be very beneficial and energising, and therefore continue to practice them. The naturopathic self-help strategies taught by the nursing staff during the day clinic, were also very well received. Almost everyone practises these in everyday life. Patients particularly enjoy applications such as dry brushing, wraps, and compresses with aromatic oils (e.g., lavender for relaxation), and water/dew treading.

Although some patients had already adopted a healthier diet prior to attending the day clinic, the lectures on the importance and benefits of a plant-based diet were additionally useful. They, for example replaced meat and sausage with whole foods, nuts, and healthy oils. The practical instructions on how to prepare healthy meals in the training kitchen, motivated many to initiate or continue with the change in nutrition. Patients have observed the simplicity of preparing tasty meals within a plant-based diet and would like to continue with it in the future.

### 3.6 Helpful and hindering aspects for implementation

All patients reported that they have implemented lifestyle changes due to participation in the day clinic. According to patients, scheduling a fixed timeslot in their daily routine for the implementation of techniques or exercises is most helpful.

Due to professional and family obligations, as well as reduced resilience, a short duration of an exercise is also considered advantageous. It was also considered important that the exercises do not require much preparation and that no second person is needed to carry them out. Having an audio guide, such as the one provided in the current program, was also considered helpful for doing the exercises at home.

The following were seen as hindrances to implementation: a high workload, job and family obligations, a general lack of time, and a lack of motivation and willpower. However, some patients admit that if you really want to, you can find the time, suggesting that the latter reasons are actually the main ones for not exercising. Some patients cited physical limitations such as joint pain during yoga exercises, as a reason for not performing exercises at home.

*“Well, these are all techniques that you can easily do on the sofa, on the bed, on the floor, so the local conditions should be there for everyone, so I don't see any problems there. And if it's important to you, I'm going to say that you take the time to do it, then you just turn the TV on for half an hour less in the evening, right? But of course, I'm going to say that I still have the advantage that I go home after six hours and, as I said, I'm home at 2:30 p.m. and can take the time. Of course, if I were to work full-time now and maybe work until 6 p.m., it would probably be too much of a good thing.” (P 5, female, 51 years)*

Overall, the satisfaction with participation in the program was very high and nearly all patients would recommend it to other PCS-patients. Patients rated the treatment very positively: they liked the multimodal approach, the variety of therapy elements and techniques, the care provided by therapists and the exchange with like-minded people. No patient discontinued the study prematurely because the intervention (participation in the day clinic program) did not meet their expectations or was too exhausting.

The perceived positive effect of the therapy is also evident from the fact that patients want to continue using the techniques they have learnt in their daily lives. Since the intervention’s explicit objective is to encourage lifestyle modifications to continue beyond the end of participation in day clinic, this can be considered to have been achieved, at least in the short term.

### 3.7 Whole-body hyperthermia

As part of the 11-week day clinic program two WBH-treatments were scheduled on separate dates. This thermo-therapy application is only offered in a few clinics or specialized medical centers in Germany. Therefore, it was a new and unfamiliar procedure for almost all of the participating patients; only one patient had previously experienced WBH during an inpatient stay.

All study participants welcomed the option of experiencing WBH as a form of therapy and initially approached the treatment with a positive attitude. Despite finding the treatment very exhausting and stressful (many did not feel well immediately afterwards, and some still felt exhausted, tired and listless the next day), the majority (14 out of 20) retrospectively reported *positive effects*: they felt as though something had been triggered in their body. Most saw positive effects on their energy levels (more energy, higher performance), but positive effects were also reported on pain intensity and “brain fog.”

*“What was also a leap forward for me, purely from a physical point of view, I clearly noticed that every time I had hyperthermia. So, I thought that was very, very good. I also found that, I mean on those days I didn't feel so good of course, but then, two days later at the latest, I always really noticed a clear improvement, that I had more energy.” (P 5, female, 51 years)*

*“But I really have the feeling that these two hyperthermia treatments have simply stabilized my state of health significantly, especially in terms of my energy levels. So, this fatigue is definitely no longer as dominant a problem as it was at the beginning or even before the day clinic.” (P 8, female, 39 years)*

Three patients perceived no effects as a result of WBH, while three other patients experienced a deterioration in their overall condition as a result of the treatment. They reported that WBH led to a recurrence of symptoms such as shortness of breath, cold symptoms or pain or even temporarily reactivated previous conditions like conjunctivitis or shingles.

Overall, the patients expressed a favorable opinion of hyperthermia as a treatment option and even those who did not benefit were satisfied to have had the experience.

## 4 Discussion

To our knowledge, the underlying mixed-methods project is the first randomized clinical trial with waiting control group to investigate the effects of an 11-week MICOM day clinic treatment on the symptoms and quality of life of Post-COVID patients in Germany. The aim of the qualitative sub-study as part of the mixed methods approach was to shed light on the individual perspective of the participants. It primarily aimed at gaining more detailed insights into the symptoms and limitations experienced by the patients, the impact of the disease on their everyday lives, the perception of the medical care provided by their treating physicians, and the extent to which they perceived changes as a result of participating in the day clinic.

In our view, the qualitative sub-study yielded the following important findings:

Firstly, Post-COVID syndrome presents with a variety of symptoms and has a significant impact on patients’ lives but has also implications at the societal level, since it affects patients’ personal lives, social interactions, activities, and professional performance. Secondly, the care provided by GPs and the treatment options offered are considered inadequate. Patients often felt that they have to take an active role in searching for suitable therapies themselves. Thirdly, the MICOM day clinic program based on a multimodal integrative medicine approach seems to be a promising approach, as it has effects on various levels and can improve symptoms and overall well-being of patients with Post-COVID syndrome. Using WBH appears to provide additional therapeutic benefits for the majority of patients.

### 4.1 Impact on personal life, healthcare system and society

The composition of our sample and the reports from participating patients on their symptoms in our study are consistent with current findings on Post-COVID syndrome in literature: the proportion of women in our study is relatively high at 85%, which corresponds to the literature describing a higher prevalence among women (9, 61). Furthermore, women are more likely to use complementary and integrative medicine (62). The clinical picture is characterized by various symptoms and, in severe cases, a deterioration in health condition after exertion called PEM (6, 10, 29). The interviews revealed that PCS has a significant impact on the daily lives of the participating patients and is associated with reduced quality of life. A substantial impact on different health domains and greatly impaired health-related quality of life in PCS and ME/CFS patients was also found by Weigel et al. (17, 63) in a cross-sectional study in Australia and in a systematic review of 16 studies from various countries worldwide. The limitations and severity of the impairment in PCS patients strongly resemble the clinical picture of ME/CFS (17). In our study, most patients reported being unable to perform normal activities at home or at work, and having to rely on the support of family and colleagues. Even worse, this condition often lasts for months or even years. In a case–control study with PCS-patients aged 18–65 in Germany there was predominantly no improvement of symptoms such as fatigue, exercise intolerance and cognitive deficits after one year of illness with no specified treatment being allocated (16).

The fact, that neither patients nor GPs know, how long convalescence will take, leads to great uncertainty and feelings of powerlessness and helplessness for patients (64). So, the finding, that patients in our study experienced an increase in self-efficacy could help to counteract the feeling of helplessness. Participating in an integrative day clinic can apparently empower patients to actively do something for their well-being and use the therapy elements they have learned, such as stress reduction techniques. They also have learned a lot about the connections between mind and body and can apply this knowledge.

For some patients, the financial consequences of the illness due to reduced working ability additionally represent a major burden. This was also evaluated in an umbrella review by Nittas et al. (65) who found that around half of the studies reported impairment in patients’ family and social lives. Some patients lose their jobs or are absent from work for long periods due to their illness. Thus, challenges would go beyond medical needs: socio-economic implications due to reduced employment or loss of employment can have negative impact on well-being and mental health. Hammer et al. (15) also report financial problems due to Post-COVID condition, which lead to reduced earning capacity leading to treatment barriers, as not all patients can afford potentially effective therapies that are not covered by health insurance (15).

The illness-related limitations are not always visible to others at first glance and by now there are no objective diagnostic markers. So, some patients feel that they are seen as malingerers who do not want to work. Fröhlich et al. (66) also emphasize this with regard to patients with ME/CFS: here, too, the etiology is unknown and the symptoms are sometimes suspected to be psychosomatic. Due to the absence of visible signs, the illness is often questioned by others (15, 66). In this context several authors highlight the risk of stigmatization (66–68). Stigmatization is a process within social relationships that devalues people based on certain characteristics. Possible consequences of stigmatization include psychological stress, anxiety and depression (67, 68). In this regard, the day clinic can provide patients with effective coping strategies. The program is designed to equip participants with the necessary skills to manage stress and anxiety effectively and enhance self-confidence (46–49). In the case of PCS, assumptions of malingering or laziness can further reduce quality of life and ability to work. Additionally, the severity of symptoms and the loss of normal functioning can make returning to work particularly challenging. Therefore, the behavior of medical professionals, employers, colleagues, and the government play a major role, as the perception of PCS as a defined illness could generate more support for patients but this has yet to happen (67). This underlines the importance of public education, which could lead to a better understanding of the disease and reduce the risk of stigmatization (66). This is all the more important since in addition to the individual consequences and limitations of the disease, there are also societal consequences. The high number of people affected and the long duration of the illness combined with a long period of incapacity to work (19) have an enormous impact on the healthcare system and society as a whole due to the associated costs. In addition, patients - many of whom require frequent medical visits, extensive diagnostics, and repeated therapy attempts (e.g., rehabilitation programs, i.e., inpatient or outpatient programs for restoring health, functionality and participation after illness) - also place a significant burden on the healthcare system. A recent study puts the expenses in Germany at several billion euros [ME/CFS report 2025 (69)]. So, the consequences of PCS are substantial for individuals and society, and the development of treatment options for this patient population is imperative to improve the quality of care and counteract the effects of the disease on society as well.

### 4.2 Therapy/medical care

As the etiology and pathogenesis of PCS are not yet fully understood and several mechanisms are assumed to play a role, no evidence-based causal therapies are available. Despite the absence of clinical markers, somatic causes cannot be excluded at present and are even probable (29). This presents a particular challenge for general practitioners (GPs), who are the primary point of contact for affected individuals and coordinate diagnostics and treatment. This situation poses a significant challenge (70). The German S1 medical guideline for Long/Post COVID (71) primarily recommends treatment based on the individual symptoms. Integrative, multimodal therapies are also recommended for the main symptoms. This is also the case for ME/CFS, which has been known for decades, and the number of people affected has doubled due to PCS (69). According to Scheibenbogen et al. (29), more research into causal therapies of post viral diseases, especially for pharmacological approaches with the aim of developing evidence-based curative treatments, is needed. Until a causal therapy becomes available, attempts must be made to treat this complex disease and its associated limitations as effectively as possible. As Lemhöfer et al. (71) also point out, an interdisciplinary approach is indicated in complex clinical pictures such as PCS, where there is no clear marker, after other diagnoses have been ruled out. Although a S1-guideline with initial treatment recommendations has been drawn up (4), the participants in our study criticize the fact that many GPs have only little knowledge of the illness and suitable treatment measures. The patients in our study were predominantly dissatisfied with the information and care provided by their GPs. Those who had already spent time in inpatient rehabilitation facilities criticized the lack of specialization. In particular, no consideration was often given to individual stress limits, so there was risk of a deterioration through activating measures (15). Hammer et al. (15) also noted in a retrospective online survey that PCS was not considered a serious illness, and that patients felt stigmatized in rehabilitation facilities due to the psychological interpretation of symptoms. Furthermore, Schmachtenberg et al. (72) showed that Post-COVID patients often do not feel taken seriously by doctors, have to wait a long time to see specialists, and the healthcare system is ill-prepared to meet their needs. Rehabilitation facilities are often not specialized in Post-COVID syndrome. Not only patients but also GPs would like more special contact points and targeted rehabilitation facilities. They are equally frustrated by the lack of causal therapies and the long waiting times for the referral of patients to specialist consultations (73). So, they often initially practice wait-and-see behavior and give general advice (e.g., taking vitamin C or D) (73) which was also reported by the patients in our study.

Of particular importance, many patients with PCS are initially too severely affected —particularly due to fatigue, pain, or cognitive dysfunction—to engage effectively in outpatient or day clinic treatment. For those with more severe symptoms, inpatient care often represents the only feasible option at the outset of the therapeutic process. In this context, structured inpatient multimodal programs offer particular promise, as they provide comprehensive, interdisciplinary interventions tailored to the complex needs of PCS patients. An initial inpatient setting can serve as a catalyst for recovery, initiating a process that fosters increasing autonomy over time. As explained above, the current treatment recommendation according to the S1-guideline is a multimodal approach, which was used as intervention in this study. The intervention in this study (MICOM day clinic) has been practiced for over 25 years for chronically ill patients and addresses all relevant lifestyle aspects (44). Paul et al. (74) assume that the application of individual therapy elements without embedding them in a comprehensive overall concept and without process support and implementation in everyday life is less effective. The program is based on the idea that patients are not passively treated, but rather actively shape their health behavior, strengthen their self-healing powers, and become active participants. Patients in our study also reported this positive effect of the day clinic: they no longer feel helpless; rather, they feel empowered to actively improve their symptoms and quality of life. In principle, the attitude toward an integrative naturopathic approach including multimodal non-pharmacologic treatment approaches in Germany is very positive: the majority - including patients in our study - already have experience with naturopathic treatments, so there is a fundamental openness. The use of complementary and integrative medical procedures in Germany is at a high level. 70% state that they have already used “Traditional, Complementary and Integrative Medicine” (TCIM), 32% have done so in the past 12 months and 18% currently. TCIM is seen predominantly as complementary therapy, not as an alternative to conventional medicine (62). An analysis of data from the NIHS of 2022 in the USA showed that almost 50% of US adults reporting Post-COVID symptoms have used complementary medicine within the past 12 months (75).

### 4.3 Effects of MICOM day clinic

The benefits from a multimodal treatment concept have been demonstrated for other chronic diseases in the area of inflammatory bowel diseases and pain disorders (i.e., fibromyalgia syndrome) before (37, 46–49, 76, 77). These illnesses have in common that they are accompanied by pain and/or fatigue, usually no causal therapy is available and stress plays an important role in the course of the disease. Using a holistic, interdisciplinary concept like MICOM in an inpatient or day clinic setting has shown to be a beneficial approach in those indication groups as well, since it can help to reduce pain and fatigue symptoms and increase quality of life.

In general, the patients with PCS who were included in this study expressed high levels of satisfaction with the day clinic and perceived positive effects. The participants reported improvements in specific symptoms, including fatigue, sleep disorders, and pain. The use of several therapy elements which are part of the day clinic in this study, namely, e.g., stress management techniques, exercise therapy and relaxation on those symptoms have been suggested as potential therapeutic measures before (22, 23, 25). In the context of lifestyle modifications, a change in diet toward a healthy, anti-inflammatory nutrition is also seen beneficial regarding PCS- and fatigue symptoms (22). Of central importance, the most significant change observed was in their attitude toward the disease. Patients reported a shift in their perception of being powerless over their condition, indicating an increased sense of autonomy and self-efficacy. Additionally, they demonstrated a more proactive approach in applying the techniques they had learned in their daily lives to reduce stress and prevent exhaustion, which can lead to a deterioration of the overall condition (4, 26). Patients further reported an increase in confidence and a decrease in anxiety, which they attributed to their improved general well-being and change in attitude. This pattern - namely, the amelioration of pain and fatigue symptoms, accompanied by predominantly attitudinal and behavioral modifications - has been previously identified in other studies conducted. The MICOM-day clinic has been shown to have a very targeted effect by training patients’ awareness of their bodies and stress symptoms and providing them with techniques for dealing with it. Individuals have the capacity to identify stress at an early stage and respond accordingly (46, 47, 78). For instance, they may employ relaxation techniques, such as breathing exercises, in high-pressure circumstances. The alterations observed among the intervention group manifested not only at the attitudinal level but also at the behavioral level. Specifically, participation in the integrative day clinic resulted in notable lifestyle modifications for a significant number of patients. This finding aligns with the outcomes of two studies on inflammatory bowel diseases, which reported alterations in lifestyle and attitudes following participation in the day clinic (46, 47). Given that the fundamental objective of the day clinic program was to enhance patient autonomy and empowerment, as opposed to merely addressing symptoms (44), it can be concluded that this objective has been accomplished. Furthermore, the attending patients reported an increase in body awareness and mindfulness. The integrative day clinic suggests a holistic approach for addressing Post-COVID syndrome, demonstrating the efficacy of the concept aimed at enhancing self-efficacy and self-care. Patients have the capacity to integrate components of naturopathic self-care measures into their daily routines, thereby assuming a more proactive role in the management of their illness.

In addition, during the MICOM-day clinic in Bamberg two treatment sessions of WBH from the field of physical therapy were offered to PCS patients. The majority reported positive benefits from using WBH, particularly an increase in their energy levels. Former studies on other chronically ill patients, e.g., patients with fibromyalgia syndrome have shown positive effects on pain (37). However, the treatment can be very strenuous for patients and must therefore be applied carefully to PCS patients so as not to overtax them. In this context, ensuring adequate stimulus intensity is crucial for therapeutic success. Only a few patients in the intervention group reported, that the treatment had a negative effect on them, which might indicate that immune processes in the body possibly triggered by the treatment led to a deterioration in general well-being. Therefore, this is probably a treatment option that did not appear to be suitable to everyone. In a retrospective analysis of data from PCS patients (*n* = 46) potentially positive effects of WBH on functional assessment and fatigue were found (42). In the mentioned study, WBH was also part of a multimodal therapy, therefore positive effects cannot be exclusively attributed to WBH and the authors excluded patients with aversion to heat treatment from the therapy. An examination of hyperthermia, in a randomized controlled trial (as currently performed by our workgroup), could provide additional insights into the effects of WBH on PCS patients without integration into the integrative-medical naturopathic concept.

An additional therapy element of the day clinic is exercise therapy (endurance and strength), which requires special caution when used in patients with PCS (26). A subset of those patients suffers from a severe form of PCS combined with PEM which means that they can experience deterioration after physical or mental exhaustion. Exercise therapy must be used with caution and the individual’s capacity and exercise limit must be considered in order to avoid post exertional malaise (4, 26). Exercise therapy can be beneficial but needs to be adapted to individual resilience and performance level (79, 80). The perceived increase of body awareness through participation in the day clinic can help patients to find out their individual limits so they can better adjust their energy management and movement units to it, in order to prevent PEM. Renz-Polster and Scheibenbogen (20) also emphasize the importance of proactive energy management as a central component of PCS treatment. In addition, to address the various manifestations of the Post-COVID condition, it is also considered crucial to implement stress management and psychosocial support measures. Furthermore, symptom-oriented treatment of pain, sleep disorders, orthostatic intolerance and depression, if applicable, is recommended as part of a comprehensive treatment strategy. This goes in line with the recommendations of EUROMENE for treatment of ME/CFS (22).

In summary, the MICOM day clinic’s holistic approach and conceptual framework have been shown to combine a multitude of elements that are currently endorsed for the treatment of Post-COVID syndrome. This integrative multimodal approach covers a wide range of essential domains in life. The program is designed to address the multifaceted needs of patients suffering from Post-COVID syndrome by incorporating various therapeutic components, including mindfulness-based stress reduction, relaxation techniques, nutritional guidance, physical exercise regimens, physical therapy interventions, and naturopathic self-help strategies. This comprehensive approach is intended to enhance patients’ self-efficacy, alleviate symptoms, and improve their overall quality of life.

### 4.4 Limitations

As this is a qualitative study focusing on the subjective perspectives of patients and based on the analysis of 20 interviews, the results cannot be generalized to all PCS patients, especially since the proportion of women in our sample is also very high. This is in addition particularly relevant given the heterogeneous nature of the clinical picture and individual situations of patients, which could not be fully represented in this sample, as specified by the RCT. For example, patients with severe fatigue symptoms who were not able to attend the day clinic program (six hours a day over a period of 11 weeks) were not included in our sample. Another limitation is, that the interviews took place quite shortly after the day clinic program had ended, so it is unclear whether the changes in symptoms and attitudes or lifestyle mentioned show long-term effects. Furthermore, this interview study did not record any effects that may have occurred at a later date following participation in the day clinic program. Moreover, it cannot be ruled out that general memory problems as a result of the retrospective questioning during the interview caused recall biases. Additionally, the holistic nature of the therapy concept means that effects cannot be attributed to individual therapy modules.

## 5 Conclusion

Five years after the start of the SARS-CoV-2 pandemic, the global consequences of the ongoing late effects of the infection - specifically Post-COVID syndrome - are being widely felt. The consequences of the illness are substantial, both on the individual and societal level. To date, there is no causal therapy anchored in a medical treatment guideline. The presented multimodal treatment concept which is based on integrative medicine and naturopathy could help to alleviate symptoms such as fatigue, improve well-being, and enhance self-efficacy. Further research into the causes of the disease and effective therapies is needed.

## Data Availability

The datasets presented in this article are not readily available because the qualitative data cannot be shared publicly due to data protection reasons and ethical considerations, because they contain sensitive information that could compromise the privacy of study participants. Requests to access the datasets should be directed to jost.langhorst@sozialstiftung-bamberg.de.
