# Supplementary material for: Integrative multimodal treatment approach for patients suffering from Post-COVID syndrome: a study based on qualitative interviews with individuals participating in an 11-week day clinic program
Source: Front Public Health. 2025 Dec 3;13:1688592. doi: 10.3389/fpubh.2025.1688592 (PMC12708604; doi:10.3389/fpubh.2025.1688592)
Supplement: Supplementary file 1 [file Data_Sheet_1.pdf]

Supplement 1. In- and exclusion criteria

Inclusion criteria	• patients between 18 and 75 years of age<br>• documented diagnosis of Post-COVID Syndrome<br>• presence of fatigue with or without myalgia.<br>• signed informed consent
Exclusion criteria	• presence of contraindications for hyperthermia (e.g., severe cardiovascular diseases, tumor diseases, acute infections, pregnant and breastfeeding women)<br>• acute and/ or febrile microbial infections<br>• pleuritic chest pain<br>• relevant shortness of breath<br>• condition after critical illness or intensive medical care due to COVID 19<br>• severe somatic, cardiovascular, pneumological, rheumatic, endocrine or neurological concomitant diseases, in particular neurological diseases associated with cognitive disorders, severe liver or kidney diseases
Suitability assessment	All patients are checked for suitability to participate in the study. If the inclusion criteria are met and there are no exclusion criteria, the patient can be included in the study.
